# Preoperative Neurological and Neurophysiological Assessment of Patients with Idiopathic Scoliosis Treated or Not Treated with Physiotherapy: A Retrospective Comparative Study

**DOI:** 10.3390/brainsci16070674

**Published:** 2026-06-27

**Authors:** Matylda Witkowska, Juliusz Huber, Tomasz Kotwicki

**Affiliations:** 1 Department of Pathophysiology of Locomotor Organs, Wiktor Dega Orthopaedic Institute, Poznań University of Medical Sciences, 28 Czerwca 1956 No 135/147, 61-545 Poznań, Poland; matylda.wit@gmail.com; 2 Department of Spine Disorders and Pediatric Orthopaedics, Wiktor Dega Orthopaedic Institute, Poznań University of Medical Sciences, 28 Czerwca 1956 r. Street, No. 135/147, 61-545 Poznań, Poland; kotwicki@ump.edu.pl

**Keywords:** idiopathic scoliosis, preoperative neurological and neurophysiological assessment, electromyography, electroneurography, motor evoked potentials, physiotherapeutic treatment, prerehabilitation

## Abstract

**Highlights:**

**What are the main findings?**

- Neurological tests supported by selected clinical neurophysiology studies reveal motor deficits and sensory abnormalities in patients with idiopathic scoliosis, especially those with Lenke 3-type rather than Lenke 1-type curvatures.
- Patients who received only bracing demonstrated poorer neurological and neurophysiological outcomes than those who received additional physiotherapy. Differences between groups treated and not treated with physiotherapy were observed; causality cannot be established, but the findings are consistent with a possible benefit.

**What are the implications of the main findings?**

- Knowledge of the right–left asymmetry of paraspinal muscle function and the weakness of specific muscle groups in the lower extremities of patients with idiopathic scoliosis can be applied in a physiotherapy strategy to compensate for the weakened muscle motor units by unilaterally increasing the number of repetitions of strengthening exercises.
- Comprehensive neurological and neurophysiological findings IS patients can help plan the treatment before spine surgery using corrective instrumentation.

**Abstract:**

**Background/Objectives**: The aim of this study was to precisely characterize neurological deficits in patients with idiophatic scoliosis (IS) by comparing preoperative clinical and neurophysiological examination results in patients with Lenke 1 and 3 spinal curvatures. Bracing alone (NTP) is commonly applied preoperatively in subjects with IS, but incorporating the concept of prerehabilitation with additional physiotherapy (TP) may further slow the progression of scoliosis. **Methods**: An interview regarding the development and conservative treatment of IS, clinical neurological assessment, and bilateral neurophysiological tests involving electromyography (sEMG) of motor unit activity in the paraspinal and lower extremity muscles, electroneurography (ENG) of neural impulse transmission in the peroneal nerve motor fibers and entire efferent conduction involving recordings of motor evoked potentials (MEPs) induced with transcranial magnetic field stimulation (TMS) were performed in patients with Lenke 1 (N = 33) and Lenke 3 (N = 27) spine curvatures in two groups (N = 30 each) that were treated (TP) or not treated (NTP) with physiotherapy. **Results**: Back pain, assessed on the VAS by all Lenke 3 patients, was 3.3 on average. Limited spine mobility (*p* < 0.001) was not associated with better results following physiotherapeutic treatment in either Lenke patient group. Sensory perception studies within the L3–S1 dermatomes and vibration sensation tests were found to be slightly decreased in Lenke 3 patients (*p* < 0.001), predominantly on the concave IS side, but less so in the TP group. Achilles tendon and patellar reflexes were detected as pathological (*p* < 0.001) only in Lenke 3 patients, and less frequently in the TP group. Asymmetry on the concave side of scoliosis in manual muscle testing scores was found (*p* < 0.001) in Lenke 3 patients, showing moderate muscle weakness in the distal lower extremities, mainly in the NTP group. sEMG recordings from the paraspinal muscles revealed moderate neurogenic abnormality that was more intense on the concave side of scoliosis curvature, both main and second; the pattern of muscle motor unit activity in the proximal and distal muscles of the lower extremities was consistent with the muscle strength deficits observed in manual muscle testing, though less so in the TP group. Deficits in MEP amplitudes recorded from lower extremity muscles and the peroneal nerve were found to be more commonly expressed on the concave side of the main scoliosis curvature and on the concave side of the second scoliosis curvature, particularly in Lenke 3 patients, but the significance of changes was lower in the TP group (*p* = 0.03–0.009). ENG studies showed moderate abnormalities in peripheral neural conduction of peroneal nerve motor fibers originating at the L5 ventral root, especially in Lenke 3 patients from the NTP group. **Conclusions**: Neurological diagnostic tests, supported by selected clinical neurophysiological studies, reveal greater motor and sensory abnormalities in IS patients with Lenke 3 than with Lenke 1 curvatures. The study indicates that patients in both groups who received only bracing had poorer outcomes than those who received additional physiotherapy. In the context of prerehabilitation, a combined conservative treatment approach including physiotherapy can provide functional benefits for the IS patient before the necessary surgical treatment. In this study, differences were observed between the groups treated with physiotherapy and those not treated; however, a causal link cannot be established. The results are consistent with a possible benefit of the physiotherapy, but they require further prospective studies to be proven.

## 1. Introduction

Idiopathic scoliosis (IS) is a disease involving three-plane spinal deformity, including vertebral rotation in the transverse plane, a tendency toward flattening of the thoracic kyphosis, and lateral deviations [1,2]. IS affects 2–3% of the population [3]. It is defined as a lateral curvature with a Cobb angle ≥ 10°, with an element of trunk rotation [4]. The current standard for assessing IS types is Lenke classification [5,6]. The familiar incidence of IS between mothers and daughters is estimated at 33% [7], and the origin of scoliosis is most often explained as multifactorial [8,9]. Lack of treatment during the development of this disease may lead to deep psychosocial consequences [10]; pathological changes in the skeletal, pulmonary, and cardiovascular systems; and, later, changes in the structures of the central and peripheral nervous systems [11,12,13]. It is, however, very often considered that primary impairments in the nervous system at the supraspinal level are the source of IS development [3]. Asymmetry in the ventral pons or medulla in the area of the corticospinal tract passage or enlarged cisterna magna has been detected in IS cases [14]. Pontine hypertrophy and asymmetry in diffusion tensor imaging metrics of the corticoreticular pathway suggest a central subcortical interhemispheric imbalance in IS [15]. There is disturbed mean diffusivity integrity along the spinal cord in girls with IS, associated with abnormal somatosensory-evoked potential (SEP) recordings which occur only above the C5–C6 levels [16]. Significant differences in the function of corticospinal tracts, which are responsible for descending motor control, have also been demonstrated in patients with idiopathic scoliosis compared to healthy individuals [12]. In patients with IS, paired-pulse TMS (transcranial magnetic stimulation) studies have also revealed reduced cortico-cortical inhibition and asymmetry in motor cortex excitability. This asymmetry was related to the side of the curvature. On the convex side, inhibition was nearly normal, whereas on the concave side, it was significantly reduced [17,18]. Pre- and postoperative TMS comparative studies in cases of patients with surgical scoliosis correction surgery have shown that MEP amplitudes increased after the procedure in the majority of girls with IS [11]. Improved efferent spinal cord neural transmission was also observed during MEP neuromonitoring after TES (transcranial electrical stimulation) in more than 30% of surgically treated girls with scoliosis [12,13].

The treatment of choice for IS is conservative, with physiotherapy and bracing [2,19,20]. These procedures may slow progression rather than reduce the pathological curvature angle. IS is diagnosed based on a clinical examination and its correlation with imaging tests [9]. A lateral Cobb’s angle of more than 40 degrees on X-ray is essential for deciding on surgical intervention for pathological curvature [21], although spine surgeons widely support simultaneous neurological assessment in patients with IS [22]. PSSE (physiotherapy-specific scoliosis exercise) stabilizes or reduces scoliosis progression in 60–80% of patients, while bracing does so in 70–90% [23]. A combination of both methods achieved an 80–90% success rate in mild and moderate cases of IS advancement, with a significant improvement in quality of life. Effectiveness depends on the degree of scoliosis, the patient’s age, and the regularity of the applied treatment [24,25,26,27]. The effectiveness of physiotherapy has been mainly evaluated through clinical methods, and neurophysiological assessment has been rarely used for this purpose [26].

Neurological symptoms of sensory and motor function abnormalities in IS patients have not been described in detail [13]. Some researchers claim that pathologies can be clearly observed only with significant advancement of scoliosis lateral curvature [22], while others state that women with scoliosis often present neurological deficits [28].

The clinical tests that are most often utilized to support the neurological evaluation of IS patients’ health status include abnormalities detected in radiographic findings [2,29] and posture impairment evaluations, such as rib hump and lumbar ridge in Adam’s test [30]. The incidence of severe back pain > 5 on the Visual Analog Scale (VAS) and radiculopathy frequency have been estimated to occur in 66% and 47% of patients, respectively [18], while increased muscle tension measured as a 2–4 on the Modified Ashworth Scale (MAS) has only been described in IS patients with neuromuscular conditions [31,32]. In general, the current available literature suggests the necessity of using more precise clinometric methods, like clinical neurophysiology tests, to define the neurological symptoms of IS patients before surgeries are applied [2,9,11,22].

The primary aim of this study is to precisely characterize neurological deficits in IS by comparing preoperative clinical and neurophysiological test results in patients with two types of spinal curvature: Lenke 1 and 3. The current characteristics of neurological assessment appear insufficient in the literature. Although bracing treatment alone (NTP) is common preoperatively in patients with IS, incorporating the concept of prerehabilitation with additional physiotherapy (TP) may possibly delay the progression of spinal curvature. Moreover, this dual approach may better prepare IS patients for the effects of intraoperative interventions and shorten the postoperative care period. We assume that, due to greater spinal deformity in the main thoracic and second thoracolumbar curvatures, patients with Lenke type 3 will have worse neurological and neurophysiological outcomes than those with Lenke type 1.

To our knowledge, this is the first evaluation of the efficiency of TP in IS Lenke 1 versus Lenke 3 patients, using available clinical assessment methods supplemented by expanded neurophysiological testing.

## 2. Materials and Methods

### 2.1. Subjects, Study Design, and Treatment Strategy

**Figure 1:**
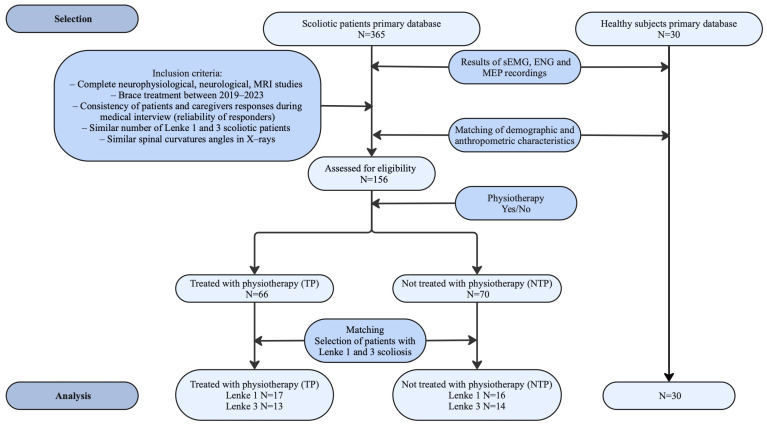
Study design, including selection criteria for participants with scoliosis and subjects included in the control group.

Data for this retrospective research were collected during routine diagnostic preoperative evaluations of patients treated for IS at the Wiktor Dega Orthopaedic and Rehabilitation Clinical Hospital in Poznań, Poland. Ethical considerations were consistent with the Declaration of Helsinki. The Bioethics Committee of the Poznań University of Medical Sciences approved the study (including research involving healthy individuals), Decision No. 942/21. Each participant (and their parent/legal guardian) was informed of the study’s purpose and provided written consent for participation in the study and publication of the data. Originally, a cohort of 365 patients with diagnosed idiopathic scoliosis was selected from the primary database, along with 30 subjects examined to assess normative values (Figure 1).

The inclusion criteria for the treatment groups in the study were conservative treatment of all IS patients with the Cheneau brace, as well as, in some patients, physiotherapy, performed between 2019 and 2023, aiming to enroll a similar number of patients with Lenke 1 and Lenke 3 scoliosis, similar spine curvature angles (main and second) on X-ray, and a complete set of neurological and neurophysiological test results. Special attention was paid to the similarity and reliability of responses among patients and their supervisors during the treatment history questionnaire. For healthy subjects, the inclusion criteria were the results from a neurophysiological evaluation using the same methods as those applied to patients and matching demographic and anthropometric characteristics with those of IS patients. After assessing eligibility for N = 156 patients, they were divided into a physiotherapeutically treated (TP, N = 66) and a non-physiotherapeutically treated (NTP, N = 70) group. The final stage was patient selection based on spine curvature type, with 30 subjects assigned to each group. The number of subjects in each group was equal to the number of healthy volunteers in whom normative values for clinical neurophysiology tests were recorded. As the neurophysiological studies relied on magnetic field and electrical stimulation, exclusion criteria for all participants included epilepsy, cardiac disease, intracranial electrodes, vascular clips or shunts, and pacemakers or other implanted biomedical devices [33]. Additionally, patients should not report head injury, stroke, mental disorders, oncological episodes, inflammatory diseases and peripheral neuropathies (verified in electroneurographic studies), and post-COVID-19-related symptoms. Based on the interview results, patients who admitted to not following medical recommendations were excluded from the study group to avoid false-positive results in the statistical analysis. All patients have been finally surgically treated for scoliosis correction using Nova Spine corrective instrumentation and have undergone intraoperative neurophysiological neuromonitoring.

The data in Table 1 indicate that girls in the patient groups and healthy subjects did not significantly differ in their anthropometric characteristics. The exceptions were minor differences, mainly in age and height, in the TP group of patients, which were unavoidable, considering the basic correspondence of the main and second curvature angles as the primary selection criterion.

### 2.2. Patients’ Medical History and Subjects’ Clinical Studies and Physiotherapy

The study protocol for the clinical trial included a set of tools for sensory and motor function evaluation, along with a medical history questionnaire. The questionnaire was designed to gather information about the onset of scoliosis (age in years), the main and second angles of the spine curvatures (main and second Cobb’s angles in degrees) measured from X-rays, the duration (in years) and frequency of bracing treatment (days per week), and the duration (in years) and frequency of certain physiotherapeutic procedures (days per week). The next part of the study involved assessing the function of the muscular and nervous systems in all subjects using selected components of a standard neurological examination. This included the assessment of pain intensity using the Visual Analog Scale (0–10, VAS), spine mobility using the finger-to-floor test (with a normal value below 10 cm) [34,35], the symptom of sciatica using the Straight Leg Rise Test—SLR, a L3–S1 dermatome sensory perception von Frey filament test (0—analgesia, 0.5—decreased, 1—normal, 2—hyperalgesia), Achilles and patellar tendon reflexes (0—absent, 1—diminished, 2—normal, 3—slightly hyperactive, 4—hyperactive with clonus), manual muscle testing (MMT; strength with Lovett’s scale: grade 5—normal, grade 4—good, grade 3—fair, grade 2—poor, grade 1—residual, grade 0—no muscle contraction), and sensation of vibration using a tuning fork [13,35]. All tests were performed by the same team of experienced physiotherapists, neurophysiologists, and surgeons. They did not know each other’s detailed interview, clinical, and neurophysiological examination results until the spreadsheets were created prior to statistical analysis. All the patients described in this paper were treated with the Cheneau brace [19]. Patients in the TP group received the elements of chosen physiotherapeutic procedures, whose principles are presented in Table 2. The most frequent procedures applied to the IS patients from the TP group were DoboMed, FITS, BSPTS, SEAS, and Schroth and Lyon.

The primary outcomes in neurological studies were results of VAS, sensory perception studies with von Frey’s (Semmens–Weinstein) filaments, and MMT; the secondary outcomes were results of X-rays, sensation of vibration, Achilles and patellar reflexes, and functional biomechanical tests, while the exploratory outcomes were the results of the patient’s medical history of scoliosis development.

### 2.3. Neurophysiological Studies

**Figure 2:**
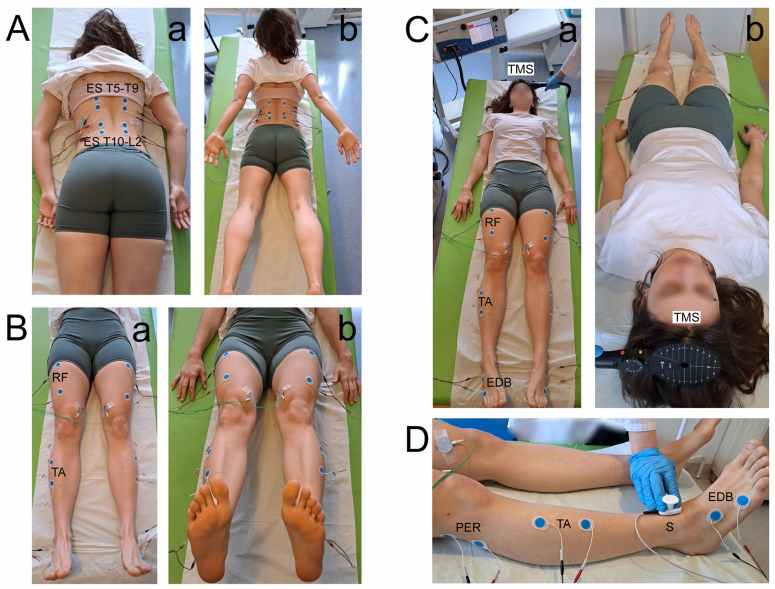
Photographs illustrating the methodology of the sEMG (**A**,**B**), MEP (**C**), and ENG (**D**) recordings, with pairs of electrodes placed bilaterally over the surface of the back and lower extremity muscles (**A**(**a**),**B**(**a**)) in healthy volunteers and in patients with scoliosis. sEMG recordings were recorded during an attempt of maximal muscle contraction lasting 5 s (**A**(**b**),**B**(**b**)). Following TMS with the coil placed over the skull (**C**(**a**,**b**)), MEP recordings were recorded bilaterally from RF, TA, EDB muscles and the peroneal nerve (**D**) over its anatomical passage at the knee ((**D**), left part). Neural transmission of the peroneal nerve motor fibers was verified in bilateral ENG recordings from EDB ((**D**), right part) following its electrical stimulation at the ankle (s). Abbreviations: sEMG—surface electromyography, ES—erector spinae, RF—rectus femoris, TA—tibialis anterior, EDB—extensor digitorum brevis, TMS—transcranial magnetic stimulation, MEP—motor-evoked potential, PER—peroneal nerve, ENG—electroneurography, “s”—stimulation.

Figure 2 presents the methodological aspects of the neurophysiological studies, both in healthy subjects and in IS girls, using the KeyPoint Diagnostic System (Medtronic A/S, Skøvlunde, Denmark). The studies were performed once in an air-conditioned room with a controlled temperature (22 °C), in a prone or supine position. In all neurophysiological measurements of the subjects, disposable Ag/AgCl surface electrodes (active surface area of 5 mm^2^) were used. The active electrode of the bipolar pair was placed on the muscle’s belly, and the reference electrode was placed on the distal tendon of the same muscle; the ground electrode was placed nearby in accordance with the Guidelines of the International Federation of Clinical Neurophysiology—European Section [44,45,46].

The first part of the examination protocol consisted of non-invasive surface electromyography (sEMG) recordings of bioelectrical activity performed bilaterally from the paravertebral erector spinae (ES) at T5–T9 and T10–L2 and the rectus femoris (RF), tibialis anterior (TA), and extensor digitorum brevis (EDB) muscles (Figure 2A,B). The sEMG signal reflects the summation of the action potentials of all muscle fibers generated during a maximal 5 s contraction to assess the functional properties of the motor units. Surface EMG recordings were always performed in patients at 60–70% of their maximal voluntary contraction (MVC). Patients were asked to perform the strongest possible contraction three times, with a 1-min rest period between contractions. The recording with the highest amplitude (µV) and frequency (Hz) was selected for analysis. Outcome measures were amplitude (expressed in μV) and motor unit action potential recruitment frequency (expressed in Hz); the recording with the largest parameters was chosen for the final analysis. To ascertain 60–70% of maximal voluntary contraction (MVC), we did not rely on subjective estimation or patient self-report. Instead, we used an objective sEMG-based methodology grounded in previously validated neurophysiological relationships between muscle force (MMT, Lovett’s scale), frequency index (FI), and signal amplitude. Own-developed and previously published [47,48] frequency index (FI, 4–0) score based on calculations of motor unit action potential recruitment frequency during maximal contraction in the sEMG recording was modified and incorporated into the analysis according to previous descriptions as follows: 4 = 95 Hz, moderate abnormality (myogenic); 3 = 95–70 Hz, normal activity; 2 = 65–40 Hz, moderate abnormality (neurogenic); 1 = 35–10 Hz, severe abnormality (neurogenic); 0 = <5 Hz, no contraction. During sEMG recordings in healthy subjects, muscle activity corresponded to MMT (Lovett’s scale) grade 5, with a frequency index of approximately 90 Hz (FI = 2.9–3.0) and minimal amplitude of 450–500 µV at 90–100% MVC. However, in iSCI patients, the similar level of contraction was not achievable, as their muscle strength during MMT (Lovett’s scale) ranged 2.3–2.8, which corresponds to a frequency index of FI = 2.6 on average and a mean EMG amplitude of approximately 250 µV.

Recordings were performed at the base time of 80 ms/D and amplification of 50–1000 µV; upper (10 kHz) and lower (20 Hz) filters were set in the recorder.

Subsequently, a transcranial magnetic stimulation (TMS) examination was performed (Figure 2C(a,b)). TMS is a non-invasive procedure that can transiently excite or inhibit corticospinal activity; in this study, the aim was evaluation of neural efferent transmission at supraspinal and spinal levels within the cortico- and rubrospinal tract fibers in the lateral and ventral white matter funiculi, as well as conduction in motor fibers of the nerves to the lower extremity muscles. Motor-evoked potentials (MEPs) were induced using single-pulse transcranial magnetic stimulation (TMS, biphasic, 5 ms in duration) with a circular magnetic coil (C-100, 12 cm in diameter) placed on the scalp over the M1 motor cortex, oriented at an angle allowing stimulation of the corticospinal tract for lower extremity muscle innervation (Figure 2C(b)). They were recorded using surface electrodes from the RF, TA, and EDB muscles (Figure 2C(a)) and the peroneal nerve (Figure 2D) on both sides. A MagPro X100 magnetic stimulator (Medtronic A/S, Skovlunde, Denmark) was used to induce the MEP recording; the magnetic field stream delivered from the coil at a strength of 70–80% of the resting motor threshold (RMT; 0.84–0.96 T) excited all neural structures up to 3–5 cm deep. The outcome measures were the amplitudes, measured from peak to peak, of the single potential in µV and the latency, measured from the onset of the recorded stimulus artifact to the positive inflexion of the MEP recording. Neither patients nor healthy volunteers reported pain during stimulation; they remained conscious and cooperative throughout the procedure. The best “hot spot” stimulation site on the skull was determined from “10–20 system” calculations and compared with the location of the largest-amplitude recording along consecutive magnetic stimulation tracks. The recorder’s low-pass filter was set to 2 kHz, and the high-pass filter to 0.5 Hz. The time base of the MEP recording was 10–20 ms/D, and the signal amplification was in the range of 200 to 5000 µV. Further methodological details on MEP recordings from lower extremity muscles and peroneal nerves along their anatomical passage have been described elsewhere [12,49,50].

Finally, bilateral verification of the peripheral motor transmission in the fibers of the peroneal nerve was performed by analyzing the results of electroneurographic (ENG) recordings from the EDB muscles for M- and F-waves following stimulation at the ankle (Figure 2D). Nerve conduction studies with recordings of M-waves (CMAPs (compound muscle action potentials)) induced with electrical stimuli in the orthodromic way (Figure 2D, “s”) were performed to diagnose the occurrence of possible neuropathies along the anatomical course of the peroneal nerve to the effector, while recordings of the frequency of long-latency F-waves verified the conduction of nerve impulses in the motor fibers of the L5 ventral roots [44,46]. This study involved stimulation with square-wave electrical pulses lasting 0.2 ms at 1 Hz, with an intensity ranging from 0 to 80 mA, using bipolar electrodes. The outcome measures were amplitude (in µV) and latency (in ms) parameters for M-waves and recording frequencies for F-waves (typically no fewer than 14 during the induction of 20 consecutive positive M-wave recordings). Recordings were performed at an amplitude of 500–5000 µV/D and a time base of 5–10 ms/D; the findings were then compared with normative values recorded in healthy volunteers.

### 2.4. Statistical Analysis

The statistical software JASP version 0.19.3 (Amsterdam, The Netherlands) was used to analyze the collected data. Measurable variables are presented with the descriptive statistics as minimal and maximal values (range), mean, and standard deviation (SD). Frequency sEMG index and recorded F-wave frequencies were of the ordinal scale type, while amplitudes and latencies were of the interval scale type. The normality of distributions was evaluated using the Shapiro–Wilk test, and the homogeneity of variances was characterized using Levene’s test. The results recorded in healthy volunteers (Controls, N = 30) and patients with IS (N = 60) were compared based on descriptive statistics. Data comparison was performed with Student’s *t*-test, if the distribution was normal; if variables did not exhibit a normal distribution, non-parametric methods such as the Mann–Whitney U test or Welch test were applied. For nominal scale values, the Chi^2^ test was applied, and *p*-values < 0.05 were considered statistically significant. Cumulative data from MEP parameter recordings performed on both sides were used for comparison when creating whisker plots. Attention was paid to matching patients’ and healthy controls’ demographic and anthropometric properties, including gender, age, height, and weight. Statistical software was used to determine the required sample size using the primary outcome variables of sEMG and MEP amplitudes recorded from EDB muscles, with a power of 80% and a significance level of 0.05 (two-tailed). The mean and standard deviation (SD) were calculated using the data from the first 15 patients, and the sample size software estimated that more than 25 patients in each group were needed for the purposes of this study to obtain reliable data.

The non-parametrical Spearman’s rank correlation coefficient (r^s^) was used to demonstrate correlations between clinical and neurophysiological results. A *p* < 0.05 significance level was assumed to be statistically significant for rank correlation.

Hedges’ (g), a standardized effect size measure for the chosen sEMG and MEP am-plitude recording parameters, has been used to quantify the magnitude of the difference between the two groups of patients and healthy subjects. A bias-corrected version of Cohen’s (d), designed to avoid overestimating effect sizes in small sample sizes, has been applied, interpreted using benchmarks as small effect ≈ 0.20, medium effect ≈ 0.50 and large effect ≥ 0.80.

## 3. Results

### 3.1. Characteristics of IS in Patients Treated (TP) and Not Treated (NTP) with Physiotherapy

As shown in Table S1, in both the Lenke 1 and Lenke 3 IS patient groups, scoliosis progression onset was reported at 4 years of age on average, with no statistically significant difference in the Lenke 3 patients within the TP group. At the time of clinical evaluation, both main and second scoliotic angles were higher in the Lenke 3 group than in the Lenke 1 group (*p* < 0.001), with the main angle values being significantly lower (*p* = 0.009) in the TP Lenke 1 group. A similar 2-year period of Cheneau bracing, approximately three to four times a week, was less preferred in the Lenke 3 group (*p* = 0.04), whereas the TP patients received physiotherapy the most frequently (three times a week), for approximately 3.5 years.

### 3.2. Results from the Clinical Studies

The neurological status of patients in both groups differed significantly from that of healthy control volunteers (*p* ≤ 0.05). Back pain, assessed on the VAS by all Lenke 3 patients in the TP and NTP groups, was similarly severe (averaging 3.3) and was significantly more sensed (*p* < 0.001) than in Lenke 1 patients (mean of 1.6). In comparison with the control group, limited spine mobility (*p* < 0.001) revealed in the finger-to-floor test was not associated with better results following physiotherapeutic treatment in either Lenke patient group. Positive SLR results were not commonly detected in Lenke IS patients. Sensory perception results within the L3-S1 dermatomes using Semmens–Weinstein filaments (FvF) and vibration sensation were found to be mostly normal and symmetrical in Lenke 1 patients but slightly decreased in Lenke 3 patients at *p* < 0.001, preferably on the concave IS side; however, this finding was less pronounced in the TP group. Achilles tendon and patellar reflexes were detected as pathological (*p* < 0.001) only in Lenke 3 patients, and less frequently in the TP group. Asymmetry on the concave side of scoliosis was reflected in manual muscle testing scores, showing moderate muscle weakness in the distal part of the lower extremities (*p* < 0.001) in Lenke 3 patients, mainly in the NTP group.

### 3.3. Neurophysiological Studies

**Figure 3:**
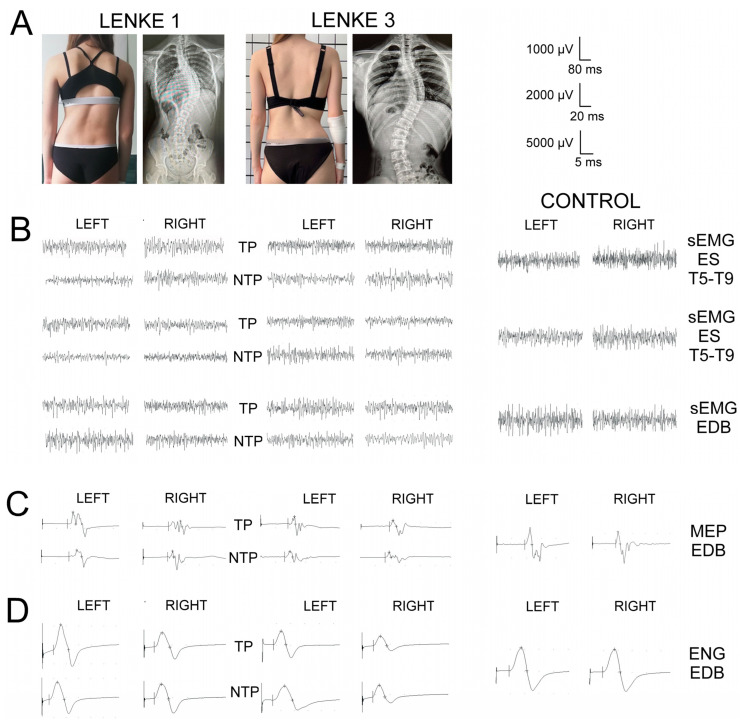
(**A**) Back views of Lenke 1 and 3 IS patients’ bodies and X-rays presenting the lateral spine curvatures. Representative bilateral sEMG recordings from Lenke 1 and Lenke 3 patients treated (TP) and not treated (NTP) with physiotherapy and from one healthy volunteer (Control) are presented in (**B**). Similarly, bilateral MEPs and ENG recordings for patients with two types of IS and a control are presented in (**C**) and (**D**), respectively. Calibration bars for amplification (vertical) and time base (horizontal) which were set during neurophysiological recordings are shown in the right upper corner of the figure. Abbreviations: sEMG—surface electromyography, ES—erector spinae, EDB—extensor digitorum brevis, MEP—motor-evoked potentials, ENG—electroneurography.

Figure 3 shows representative sample recordings from sEMG (B), MEP (C), and ENG (D) studies in patients with IS type Lenke 1 and Lenke 3 treated with physiotherapy (TP) and those not receiving physiotherapeutic treatment (NTP), as well as analogous recordings in one of the healthy volunteers from the control group for comparison.

The data in Table S2 regarding sEMG recordings in patients with IS show significant differences (*p* < 0.001) in the amplitude decrease parameter, rather than the FI frequency index, compared to the corresponding parameters recorded in healthy volunteers. In both Lenke 1 and 3 patients, these deficits in recordings from the ES T5–T9 muscles are more pronounced on the concave side of the main scoliosis curvature, as well as in recordings from the ES Th10-L2 muscles on the concave side of the second scoliosis curvature, especially in Lenke 3 patients (Figure 3B, top recordings). Overall, however, the significance of changes in parameters indicating the neurogenic nature of moderate muscle pathology is lower in both groups of patients in the TP group (*p* = 0.04–0.009). Greater abnormalities of both analyzed electromyogram parameters, suggesting neurogenic changes of moderate intensity, could be observed in sEMG recordings from more distal than proximal muscles in the lower extremities, within the range of L5 rather than L4 root innervation on the concave side of the second scoliosis curvature, mainly in Lenke 3 patients from the NTP group (Figure 3B, bottom recordings). This pattern of abnormal motor unit activity in the proximal and distal muscles of the lower extremities, as shown in sEMG recordings, was related to the muscle strength deficits observed in manual muscle testing (MMT) in Lenke 3 patients (see data in Table S1, bottom).

Analyzing the data in Table S3 on MEP recordings in IS patients, significant differences (*p* < 0.001) were found—mainly a decrease in the amplitude parameter rather than an increase in the latency parameter, compared to normative recordings in the control group. In both Lenke 1 and 3 patients, these deficits in recordings from lower extremity muscles and the peroneal nerve along its anatomical passage at the knee are more pronounced on the concave side of the main scoliosis curvature and the concave side of the second scoliosis curvature, especially in Lenke 3 patients. The significance of changes was lower in both TP groups (*p* = 0.03–0.009). The decrease in MEP amplitude rather than the increase in latency parameters of MEPs recorded from TA and EDB muscles (Figure 3C), as well as the peroneal nerves, with an increase in main and second Cobb’s angles, was more clearly observed in patients with Lenke 3 curvature than in those with Lenke 1.

Peripheral conduction studies of orthodromic neural transmission in the peroneal nerve motor fibers (Table S4, ENG) revealed moderate axonal changes, as evidenced by reduced amplitude parameters of the recorded M-waves in patients compared with normative values in healthy volunteers (*p* < 0.001). This phenomenon was less pronounced in Lenke 1 than in Lenke 3 patients; TP groups showed amplitude parameters closer to the reference values. The latency parameters of the recorded M-waves in patients showed a similar pattern of abnormalities recorded in patients. ENG studies of antidromally conducted F-waves following peroneal nerve stimulation showed a reduced detection rate compared to reference values at *p* < 0.001, particularly in patients with IS Lenke 3 from the NTP group on the concave side of scoliosis. All of the above-mentioned ENG data may indicate changes in the peripheral neural conduction of peroneal nerve motor fibers originating at the level of the L5 ventral root.

The correlation study results for the examined parameters recorded in the clinical and neurophysiological studies are presented in Table 3. We found very weak positive correlations between decreases in the amplitude and frequency parameters in mcsEMG recordings from TA muscles and a decrease in the MMT value scores measured from the same muscles, which was more expressed in patients from the NTP group. Moreover, a weak positive correlation between a decrease in the amplitudes in MEP recordings from EDB muscles and a decrease in the amplitudes of M-waves in the electroneurographic studies following stimulation of motor fibers in peroneal nerves was detected in the NTP group.

The results presented in Table 4 confirm the effect size of the chosen neurophysiological findings: large differences between healthy subjects and patients, and medium differences between patients belonging to both groups. Patients with Lenke 3 vs. Lenke 1 spinal curvatures who were not treated with physiotherapy differed more than those of both groups who received physiotherapy.

## 4. Discussion

### 4.1. Clinical Studies

The results presented in this report provide the first comprehensive description of the neurological status of sensory and motor impairments in IS patients aged 10–18 years using well-known clinical tests with additional reference to neurophysiological test results. Previous general descriptions of clinical tests have focused mainly on scoliotic patients across different Lenke types at various stages of disease progression. In our study, we complemented the previous observations of Daroszewski et al. [13], who assessed tibialis anterior muscle strength in Lenke 1 patients with a median score of 3–4. The incidence of severe back pain > 5 in VAS [18], in all studied Lenke 3 patients, was similar to our observation of 3.3 on average. Limited spine mobility was not associated with better outcomes following physiotherapy in all IS patients in our sample. Smith et al. [28] quantified the prevalence of neurological symptoms and deficits in adults (mean age: 63 years) with scoliosis presenting to a surgical clinic, with preoperative incidences of back pain (6.7 on the VAS) and radiculopathy of 66% and 47%, respectively. They concluded that neurological symptoms and deficits are common among adults with scoliosis. Moreover, the development of neurological symptoms and/or deficits was strongly associated with the decision to pursue operative treatment. The remaining clinical trial results presented in Table S1 are original data and therefore cannot be compared with other researchers’ results.

### 4.2. Neurophysiological Studies

**Figure 4:**
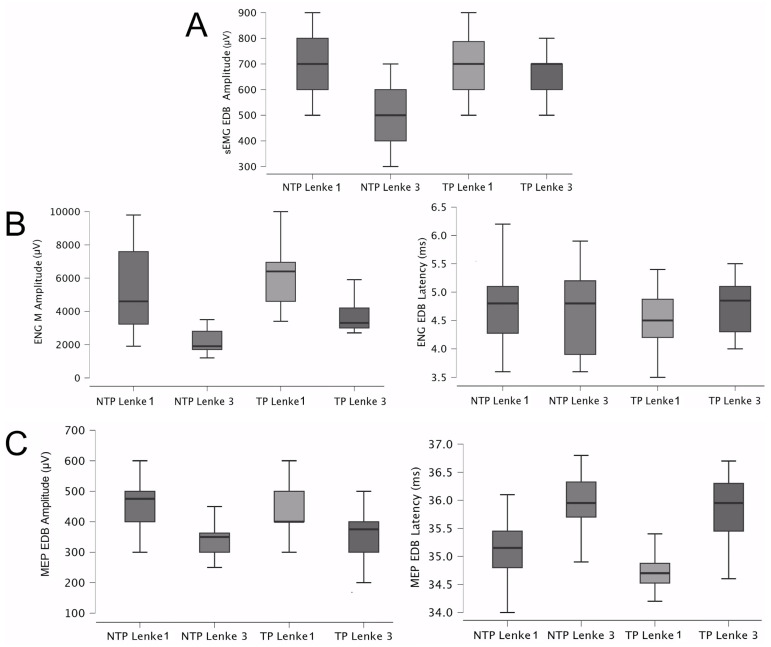
Distribution of data on amplitude (middle and right sides) and latency parameters (left sides) in sEMG (**A**), ENG (**B**) and MEP (**C**) recordings in Lenke 1 and Lenke 3 patients not treated or treated with physiotherapy. Boxes encompass the interquartile range, which represents the middle 50% of the data. The lower edge of the boxes represents the first quartile (Q1, 25th percentile). The upper edge of the boxes represents the third quartile (Q3, 75th percentile). Lines extending from the box show the variability of the data outside the middle 50%. The horizontal line inside the box represents the median (second quartile, 50th percentile), which is the middle value in an ordered data set. Abbreviations: EDB—extensor digitorum brevis muscle, sEMG—surface electromyography, ENG—electroneurography, MEP—motor-evoked potentials, not treated (NTP) and treated (TP) with physiotherapy.

The main findings from the neurophysiological tests presented in this paper can be briefly summarized with the data presented in the histograms in Figure 4, where the cumulative results refer to recordings from the extensor digiti brevis muscles during sEMG (A), ENG (B), and MEP (C) studies in patients treated with bracing alone or with physiotherapy support. A marked decrease in the amplitude parameters of electromyograms describing motor unit activity during the maximal contraction test was observed in the Lenke 3 group of patients compared to the Lenke 1 group of patients, both not receiving physiotherapy (A). A similar trend of abnormalities in amplitude parameters without significant deficits in latency parameters in M-wave electroneurographic recordings (B) suggests that the neurogenic nature of muscle damage results primarily from pathology in the motor axons of the peroneal nerves, as observed again in the NTP group of patients. To a significant extent, deficits in peripheral motor conduction contribute to decreased amplitude parameters rather than increased latency values of transcranial motor-evoked potentials (C), verifying neural transmission from the upper motor neuron to the muscles. However, considering the asymmetry of efferent conduction in the fibers of the corticospinal and rubrospinal tracts at the level of the lateral funiculi found in MEP studies in the areas of main thoracic (patients Lenke 1) and second thoracolumbar (patients Lenke 3) curvatures, changes in the peripheral nervous system may be a consequence rather than a cause of IS.

It is important to note that the results from all neurophysiological tests presented in this study were significantly asymmetric and worse on the concave side; their diversity depended on whether the observations were performed in a patient with Lenke 1 or Lenke 3 curvature. It is understandable that, due to greater spinal deformity in the main thoracic and second thoracolumbar curvatures, patients with Lenke type 3 will have worse MEP recording results than patients with Lenke type 1. The hypothesis of a neurological basis for scoliosis at the supraspinal level is possible, because even a minor neurological defect, such as impaired neuromuscular control or sensory perception, can lead to spinal curvature [51]. Abnormalities in corticospinal neurotransmission or asymmetric hyperexcitability of hemispheric motor centers [17] also contribute to the development of idiopathic scoliosis, which may explain why the Lenke 1 and Lenke 3 patterns of IS in this study might present relevant neurofunctional differences. These findings may support previous studies suggesting that the cause of idiopathic scoliosis is an abnormality of the central nervous system [4,14,15,16], as also evidenced by the abnormal amplitude parameters in motor-evoked potentials recorded in our study in a larger number of patients with IS Lenke 3.

Previous sEMG studies of paravertebral muscles in patients with scoliosis suggested higher activity of the muscle motor units on the convex side [52,53], while others found no significant differences on both sides of the scoliotic spine [54]. However, researchers have consistently emphasized that large-scale electromyography screening could help identify abnormal spinal muscle function before scoliosis is manifested. The results of our current observations confirm the neurogenic nature of muscle dysfunction of moderate severity, with a marked reduction in the amplitude and frequency parameters of electromyograms recorded from the paraspinal muscles more on the concave side of scoliosis in Lenke 1 patients in the main angle of curvature at the thoracic level, confirming the observation of Huber et al. [55] and the report by Cheung et al. [56]. The results of sEMG recording from the tibialis anterior muscles in patients with Lenke 1 curvature presented in Table S2 are consistent with the reports of Daroszewski et al. [12,13]; the present observations also extended the data on the parameters of electromyograms recorded from the lower extremity muscles in the range of L4 and L5 ventral root innervation. We also demonstrated a pattern of reversal lateralization of dysfunction on the concave side in paraspinal muscles with Th10-L2 ventral root innervation and in lower extremity muscles with L4–L5 innervation due to the significant second scoliotic angle in Lenke 3 patients.

Both results of MEP studies in Lenke 1 patients were characterized by similar parameters, albeit with slightly higher amplitudes but similar latency values to those described by Daroszewski et al. [12,13], but not Kimiskidis et al. [57] and Luc et al. [58], for recordings from the tibialis anterior muscle and direct supracutaneous recordings over the anatomical passage of the peroneal nerve at the knee. However, the ENG results of peripheral impulse conduction studies in the motor fibers of the peroneal nerve are comparable in terms of parameters of amplitudes and latencies in M-wave recordings, as well as F-wave recording frequency. The original added value described in this study is the MEP recording parameters obtained in Lenke 3 patients. However, the pattern of lateralization asymmetry—similar to that in the case of bilateral sEMG recordings from the lower extremity muscles—was similar and characteristic in both groups of patients, with a greater degree of pathology in Lenke 3 than in Lenke 1 patients.

### 4.3. Clinical Significance of the Results

Comparing neurological and neurophysiological test results according to the proposed approach may be important in making the final decision regarding IS surgery and its personalization after attempts at conservative treatment with bracing and physiotherapy. Periodic neurophysiological testing, comparing numerical parameters and enriching the classical clinical assessment, can serve as a sensitive biomarker for assessing the progress of IS treatment. To our knowledge, this study presents correlation results indicating consistency between lower extremity muscle strength tests and neurophysiological evaluation of motor neuron transmission in patients with IS for the first time. However, it is important to remember that correlations do not necessarily indicate causality, and other factors may influence the relationship between variables. Therefore, clinical decisions should be based on a comprehensive assessment of all relevant factors and individual patient characteristics, which are highly variable in patients with IS, depending on age and spinal curvature angles.

Knowledge of the right–left asymmetry of paraspinal muscle function and the weakness of specific muscle groups in the lower extremities, which differ in extent between the two types of curvatures, Lenke 1 and Lenke 3, has both cognitive and applied value in physiotherapy designed to inhibit the development of scoliosis [36]. Kinesiology can then be implemented with the aim of compensating for the contraction of weakened muscle motor units by unilaterally increasing the number of repetitions in strengthening exercises [24,25,26,59]. Such compensatory exercises help in restoring proper muscle balance on both sides, limiting and slowing the progression of spinal curvature [24,25,26,27,38]. The current approach to physiotherapy treatment assumes a high degree of individualization and the selection of appropriate exercises and treatment strategies tailored to each patient. The neurophysiological findings included in our study, particularly sEMG, may have a greater impact on the therapeutic approach in patients with Lenke 1 than in those with Lenke 3, due to the easier ability to compensate for single than double lateral curvature. Furthermore, large-scale EMG screening could help in detecting abnormal spinal muscle function before scoliosis manifests.

The spine surgeon is particularly interested in a detailed preoperative assessment of the patient’s neurological status, the results of which are generally inconsistent with neuroimaging results. Furthermore, sEMG, ENG, and MEP findings prior to IS spine surgery can be helpful in planning the scope of treatment using corrective instrumentation, where the result is not only aesthetic improvement, but also restoration of normal anatomical and functional relationships of neural structures in the spinal canal of the deformed spine, primarily through bilateral scoliosis curvature distraction and derotation procedures following implantation of two rods.

In the available literature, this is the first description of the effect of conjoined bracing and physiotherapy in IS Lenke 1 versus Lenke 3 patients, using clinical assessment methods supplemented by expanded neurophysiological testing. The presented scheme of clinical and neurophysiological examinations in patients with IS types Lenke 1 and Lenke 3 performed preoperatively, and the comparison of the results with reference values obtained in healthy volunteers—especially for the recording of motor-evoked potentials—should become a strategic starting point for the neurophysiologist planning intraoperative neuromonitoring [12,33], aimed at increasing the patient’s safety and ensuring the surgeon’s work comfort.

### 4.4. Study Limitations

One can raise an objection that the value of the presented findings can be limited by the sample size; however, as mentioned in Section 2.4, the statistical software estimated the confidence of the intended results when each group of TP and NTP subjects consists of more than 20 patients. Moreover, preliminary data mining was aimed at selecting patients in both groups with main and second scoliotic curvature angles and adjusting anthropometric data to be homogeneous.

One limitation of our study is the lack of correction for multiple comparisons in analyses involving multiple variables, which may have increased the risk of Type I error. The performed tests addressed distinct clinical questions rather than repeated testing for the same hypothesis. The pairwise analyses were predefined based on a priori hypotheses. Therefore, Bonferroni correction was not applied, as it could be overly conservative and increase the probability of Type II error in this sample. Finally, it must be noted that this study was exploratory in nature. Consequently, the statistical analyses were conducted without common multiplicity corrections to ensure that novel trends were not overlooked. While this approach served our goal of hypothesis generation, the reported associations should be interpreted with caution and require confirmatory testing with independent cohorts of IS patients.

Another problem was the trustworthiness of the answers to the medical questionnaire administered as part of the project regarding the frequency of corset use and specific physiotherapy treatments; the psychological influence on the report cannot be overestimated. However, during their conduction, special attention was paid to the similarity and reliability of responses among patients and their supervisors during the treatment history questionnaire. In cases of doubt regarding the consistency of the responses, the patient was excluded from the study design at the stage of selecting subjects for data analysis. During the report, patients and parents were assured that the answers would not affect qualification for the future surgical correction. Moreover, the onset of scoliosis and its visible progression had to be consistently reported by the parent and confirmed by the young patient. The great majority of patients used the Cheneau brace during the day, rarely at night.

The physiotherapy procedures used in this study for IS patients appear diverse, which could have influenced the final treatment outcome in the TP group. However, the aim of this study was not to verify the effectiveness of the specific physiotherapy method. All of the methods are recommended by SOSORT (see Table 2) and commonly used for the treatment of patients in Poland and worldwide.

As noted in the Materials and Methods chapter, after analyzing the data in Table 1, the statistical tool indicated slight differences in anthropometric characteristics and age between patients in the TP and NTP groups. However, from a clinical perspective, and given that sEMG recordings were always performed at 60–70% of the maximal voluntary contraction (MVC), these factors cannot significantly influence the final results, especially when comparing parameters recorded in patients and in the healthy control group. In control subjects and all patients, the average age (14.0 vs. 13.0), height (153.8 vs. 152.3), and weight (44.8 vs. 42.7) did not statistically differ. According to our previous studies using the same sEMG methodology of analyzing the amplitude parameters (in µV) and frequency of motor unit potential firing (FI index, in Hz) [12,13,46,47,48,50], the principle of recording of “the larger the muscle, the larger the signal source is” cannot influence the final results in this study.

In future research, we should exclude potential errors, especially those arising from patient selection biases related to the scoliosis curvature regarding the different Lenke types, the lack of typical randomization and therapeutic heterogeneity, and imperfectly measured adherence, which may influence the study results.

## 5. Conclusions

Neurological diagnostic tests, supported by selected clinical neurophysiological studies, reveal motor deficits and sensory abnormalities in patients with IS, especially those with Lenke 3 curvatures, rather than Lenke 1. Preoperatively, patients in both groups who received only bracing demonstrated poorer outcomes than those treated additionally with physiotherapy. The study reports a correlation between abnormal clinical muscle strength scores and neurophysiological efferent transmission test results, both in physiotherapeutically treated and untreated groups of patients, confirming these evaluations as complementary tools for motor function diagnostics in IS patients; these results require further confirmation in prospective studies.

## Figures and Tables

**Table 1:** Anthropometric data of the girls with IS and healthy volunteers from the control group. Minimum, maximum, mean value, and standard deviation are presented.

Variable Subject Groups	Age (Years)	Height (cm)	Weight (kg)
**Healthy volunteers (Control)**	11–18	122–171	35–64
**N = 30**	14.0 ± 2.1	153.8 ± 13.0	44.8 ± 11.9
**All patients NTP**	10–17	132–181	30–64
**N = 30**	13.0 ± 1.9	152.3 ± 12.1	42.7 ± 8.0
**All patients TP group**	10–17	131–179	29–59
**N = 30**	13.4 ± 1.5	153.7 ± 12.0	43.0 ± 7.1
**Lenke 1 NTP group**	10–16	140–168	31–54
**N = 16**	12.9 ± 1.6	154.3 ± 9.4	42.9 ± 6.8
**Lenke 1 TP group**	10–14	131–167	29–53
**N = 17**	12.4 ± 1.1	148.0 ± 10.6	40.2 ± 6.4
**Lenke 3 NTP group**	10–17	132–181	30–64
**N = 14**	13.0 ± 2.2	150.0 ± 14.6	42.5 ± 9.5
**Lenke 3 TP group**	13–17	145–179	40–59
**N = 13**	14.7 ± 1.0	161.2 ± 9.6	46.6 ± 6.4
***p*-value** **(difference)**			
		
Control vs. All patients	0.053	0.763	0.134
Control vs. Lenke 1	**0.014**	0.346	0.054
Control vs. Lenke 1 NTP	0.087	0.911	0.332
Control vs. Lenke 1 TP	**0.013**	0.122	**0.031**
Lenke 1 NTP vs. Lenke 1 TP	0.266	0.083	0.239
Control vs. Lenke 3	0.948	0.656	0.581
Control vs. Lenke 3 NTP	0.160	0.386	0.241
Control vs. Lenke 3 TP	0.217	0.073	0.761
Lenke 3 NTP vs. Lenke 3 TP	**0.028**	**0.039**	0.065
Lenke 1 vs. Lenke 3	**0.004**	0.161	1.128
Lenke 1 NTP vs. Lenke3 NTP	0.929	0.289	0.884
Lenke 1 TP vs. Lenke 3 TP	**0.001**	**0.001**	**0.026**

Abbreviations: NTP—patients not treated with physiotherapy; TP—patients treated with physiotherapy; statistically significant differences are marked in bold.

**Table 2:** Physical therapy treatment systems whose elements were applied to the patients belonging to TP group, including the country of origin and a description of the treatment method.

Physiotherapeutic System	Country of Origin	Principles	Source
Schroth	Germany	- three-dimensional IS therapy focused on pattern-specific postural correction - core principles: auto-elongation (detorsion), deflection, derotation, rotational breathing, and stabilization - visual feedback - recalibration of normal postural alignment and static/dynamic postural control - improving spinal stability	Moramarco et al. [36] Kocaman et al. [37] Seleviciene et al. [38]
DoboMed	Poland	- emphasizes deepening thoracic kyphosis through exercises performed in closed kinematic chains - based on a symmetrically aligned pelvis and shoulder girdle, followed by active stabilization of the corrected posture - incorporates rotational angular breathing exercises	Seleviciene et al. [38] Dobosiewicz et al. [39] Berdishevsky et al. [40] Wnuk et al. [41]
BSPTS	Spain	- three-dimensional postural correction - the expansion/contraction breathing technique - stabilization through muscular tension improvement - functional integration of daily living activities	Seleviciene et al. [38] Berdishevsky et al. [40] Jelačić et al. [42]
SEAS	Italy	- autocorrection and stabilization - enhancing spine stability - addressed to any impairments identified during the initial evaluation, such as deficits in strength, muscular tightness, or motor coordination	Seleviciene et al. [38] Berdishevsky et al. [40]
FITS	Poland	- increasing the girl’s awareness of spinal deformities and guiding towards effective scoliosis correction - releasing myofascial restrictions, improving thoracic kyphosis, and aligning the pelvis through proper foot loading - strengthening deep spinal and pelvic floor muscles enhancing the trunk stability - corrective breathing, postural training, and balance exercises, applying skills to daily activities like sitting, walking, and functional movements	Seleviciene et al. [38] Berdishevsky et al. [40]
Lyon	France	- improving motivation towards bracing - patient education, including awareness of postural defects - increasing the range of motion - neuromuscular control of the spine, trunk stabilization, muscle strength, respiration, and ergonomics	Seleviciene et al. [38] Berdishevsky et al. [40] Büyükturan et al. [43]

**Table 3:** Spearman’s rank correlation (r_s_) calculated for the sEMG, MEP measurements, and MMT study results in all patients. Cumulative data from the right and left sides are presented. *p* ≤ 0.05 was assumed as statistically significant for rank correlation (marked in bold).

	NTP		TP	
Parameter	MMT TA		MMT TA	
**mcsEMG TA** **Amplitude (µV)** **Frequency index (FI 4–0)**	r_s_	*p*	r_s_	*p*
	0.267 0.212	**0.03** **0.04**	0.121 0.131	**0.05** **0.05**
	ENG EDB M-wave Amplitude (µV)		ENG EDB M-wave Amplitude (µV)	
**MEP EDB** **Amplitude (µV)**	r_s_	*p*	r_s_	*p*
	0.082	**0.04**	0.061	0.06

Abbreviations: MMT—evaluation of the muscle’s strength (0–5); TA—tibialis anterior muscle; mcsEMG—sEMG recording during maximal muscle’s contraction; FI—frequency index (4–0)—frequency of motor unit action potentials recruitment during maximal contraction sEMG recording; ENG—electroneurography; EDB—extensor digitorum brevis muscle; M-wave—compound muscle action potential recorded following electrical stimulation of peroneal nerve, MEP—motor-evoked potential; *p* < 0.05 determines significant statistical differences (marked in bold).

**Table 4:** Hedges’ g effect size calculated for the chosen sEMG and MEP amplitude measurements in healthy subjects vs. patients and between patients belonging to Lenke 1 and Lenke 3 groups.

Group of Subjects	sEMG EDB Amplitude (µV)		MEP EDB Amplitude (µV)	
	Right	Left	Right	Left
**All patients vs. Control**	1.000	0.994	1.000	1.000
**Lenke 3 vs. Lenke 1**	0.477	0.561	0.659	0.510
**NTP Lenke 3 vs. NTP Lenke 1**	2.285	2.206	1.685	1.026
**TP Lenke 3 vs. TP Lenke 1**	0.072	0.266	1.011	0.980
**NTP Lenke 3 vs. TP Lenke 3**	–0.901	–2.149	–0.071	–0.330
**NTP Lenke 1 vs. TP Lenke 1**	–0.151	0.039	0.035	–0.066

Abbreviations: EDB—extensor digitorum brevis muscle; sEMG—surface electromyography; MEP—motor evoked potential; NTP—patients not treated with physiotherapy; TP—patients treated with physiotherapy.

## Data Availability

All the data generated or analyzed during this study are included in this published article.

## Supplementary Materials

The following supporting information can be downloaded at: https://www.mdpi.com/article/10.3390/brainsci16070674/s1, Table S1: Comparison of the patients’ medical history analysis, as well as the results of the clinical studies performed in the two groups treated or not treated with physiotherapy. Range, mean, and standard deviation values are presented; *p* ≤ 0.05 determines significant statistical differences (marked in bold); Table S2: Comparison of electromyography recordings results from healthy volunteers and two groups of patients, treated or not treated with physiotherapy. Range, mean, and standard deviation values are presented; *p* ≤ 0.05 determines significant statistical differences (marked in bold); Table S3: Comparison of motor-evoked potential recording results from healthy volunteers and two groups of patients, treated or not treated with physiotherapy. Range, mean, and standard deviation values are presented; *p* ≤ 0.05 determines significant statistical differences (marked in bold); Table S4: Comparison of the bilateral electroneurography (ENG) recordings results following the stimulation of peroneal nerves in healthy volunteers and two groups of patients, treated or not treated with physiotherapy. Range, mean, and standard deviation values are presented; *p* ≤ 0.05 determines significant statistical differences (marked in bold).
