# Supplementary material for: Preoperative Neurological and Neurophysiological Assessment of Patients with Idiopathic Scoliosis Treated or Not Treated with Physiotherapy: A Retrospective Comparative Study
Source: Brain Sci. 2026 Jun 27;16(7):674. doi: 10.3390/brainsci16070674 (PMC13407046; doi:10.3390/brainsci16070674)
Supplement: Supplementary file 1 [file brainsci-16-00674-s001.zip › brainsci-4340720-supplementary.pdf]

Table S1. Comparison of the patients' medical history analysis, as well as the results of the clinical studies performed in the two groups treated or not treated with physiotherapy. Range, mean, and standard deviation values are presented; p≤0.05 determines significant statistical differences (marked in bold).

		All patients					Lenke 1 group						Lenke 3 group								
	**Healthy volunteers (Control)**	NTP	TP	All patients vs. Control *p*	NTP patients vs. Control *p*	TP patients vs. Control *p*	NTP	TP	Lenke 1 vs. Control *p*	NTP Lenke 1 vs. Control *p*	TP Lenke 1 vs. Control *p*	NTP Lenke 1 vs. TP Lenke 1 *p*	NTP	TP	Lenke 3 vs. Control *p*	NTP Lenke 3 vs. Control *p*	TP Lenke 3 vs. Control *p*	NTP Lenke 3 vs. TP Lenke 3 *p*	Lenke 1 vs. Lenke 3 *p*	NTP Lenke 1 vs. NTP Lenke 3 *p*	TP Lenke 1 vs. TP Lenke 3 *p*
**Patients' medical history**																					
**Scoliosis progression onset (age in years)**	NA	3-6<br>4.6±0.7	3-7<br>4.6±1.0	NA	NA	NA	4-6<br>4.5±0.7	3-5<br>4.0±0.6	NA	NA	NA	0.061	3-6<br>4.6±0.7	5-7<br>5.5±0.7	NA	NA	NA	**0.008**	**<0.001**	0.400	**<0.001**
**Main scoliotic angle (º)**	NA	45-84<br>59.2±12.9	41-82<br>53.2±10.1	NA	NA	NA	45-77<br>51.4±8.3	41-51<br>46.1±2.8	NA	NA	NA	**0.009**	45-84<br>68.0±11.6	52-82<br>62.6±8.1	NA	NA	NA	0.176	**<0.001**	**0.001**	**<0.001**
**Second scoliotic angle (º)**	NA	23-50<br>37.4±8.8	25-52<br>34.5±8.4	NA	NA	NA	24-40<br>31.6±5.3	25-34<br>28.9±2.6	NA	NA	NA	0.129	23-50<br>44.0±7.2	28-52<br>41.9±7.6	NA	NA	NA	0.559	**<0.001**	**<0.001**	**<0.001**
**Brace treatment (years)**	NA	0.5-3.0<br>2.1±0.9	0.5-3.0<br>1.9±0.8	NA	NA	NA	1-3<br>2.3±0.7	0.5-3<br>2.0±0.9	NA	NA	NA	0.293	0.5-3.0<br>1.8±0.1	0.5-3.0<br>1.8±0.8	NA	NA	NA	0.980	0.124	0.157	0.498
**Frequency of brace wearing (days per week)**	NA	2.0-6.0<br>3.7±1.1	1-6<br>3.3±1.2	NA	NA	NA	2-6<br>3.9±1.0	1-6<br>3.8±1.2	NA	NA	NA	0.787	2-5<br>3.5±1.1	1-4<br>2.6±0.8	NA	NA	NA	**0.042**	**0.003**	0.242	**0.002**
**Physiotherapy duration (years)**	NA	NA	2-5<br>3.4±0.9	NA	NA	NA	NA	2-5<br>3.2±0.8	NA	NA	NA	NA	0-0<br>0.0±0.0	2-5<br>3.7±1.0	NA	NA	NA	NA	0.823	NA	0.198
**Frequency of physiotherapy (days per week)**	NA	NA	1-4<br>2.1±0.9	**<0.001**	NA	**<0.001**	NA	1-4<br>2.8±1.0	**<0.001**	NA	**<0.001**	**<0.001**	0-0<br>0.0±0.0	2-4<br>3.2±0.7	**<0.001**	NA	**<0.001**	**<0.001**	0.533	NA	0.376
**Clinical studies**																					
**Back pain VAS (0-10)**	NA	0.0-4.0<br>2.4±1.3	0-4<br>2.3±0.9	**<0.001**	**<0.001**	**<0.001**	0-3<br>1.5±0.9	0-3<br>1.8±0.7	**<0.001**	**<0.001**	**<0.001**	0.706	3-4<br>3.5±0.5	2-4<br>3.1±0.5	**<0.001**	**<0.001**	**<0.001**	0.118	**<0.001**	**<0.001**	**<0.001**
**Finger-to-floor test (cm)**	0-2<br>0.2±0.5	0-3<br>1.4±1.1	0-2<br>0.7±0.8	**0.001**	**<0.001**	**0.023**	0-3<br>0.9±1.1	0-2<br>0.5±0.6	0.112	**0.023**	0.294	0.118	0-3<br>1.9±0.9	0-2<br>1.0±0.8	**<0.001**	**<0.001**	**0.004**	0.083	**0.022**	0.059	0.135
**SLR test (0 = negative, 1 = positive)**	0-0<br>0.0±0.0	0-1<br>0.4±0.5	0-1<br>0.2±0.4	**<0.001**	**<0.001**	**0.010**	0-1<br>0.2±0.4	0-1<br>0.1±0.2	0.049	0.014	0.179	0.258	0-1<br>0.7±0.5	0-1<br>0.4±0.5	**<0.001**	**<0.001**	**<0.001**	0.085	**<0.001**	**0.004**	**0.027**
**FvF index (0-2)**	1-1<br>1.0±0.0	0.5-1.0<br>0.8±0.2	0.5-1.0<br>0.9±0.2	**<0.001**	**<0.001**	**0.002**	1.0-1.0<br>1.0±0.0	0.5-1.0<br>0.9±0.2	0.171	NA	0.055	0.157	0.5-1.0<br>0.6±0.2	0.5-1.0<br>0.8±0.3	**<0.001**	**<0.001**	**<0.001**	0.182	**<0.001**	**<0.001**	**0.035**
**FvF symmetry (1 = asymmetrical 2 = symmetrical)**	2-2<br>2.0±0.0	1-2<br>1.7±0.5	1-2<br>1.7±0.5	**0.001**	**0.002**	**0.001**	2-2<br>2.0±0.0	1-2<br>1.8±0.4	0.049	NA	**0.005**	**0.038**	1-2<br>1.4±0.5	1-2<br>1.6±0.5	**<0.001**	**<0.001**	**<0.001**	0.332	**0.002**	**<0.001**	0.376
**Vibration sensation (tuning fork test index) (1 = normal, 0 = abnormal)**	1-1<br>1.0±0.0	0-1<br>0.7±0.5	0-1<br>0.7±0.5	**<0.001**	**<0.001**	**0.002**	1-1<br>1.0±0.0	0-1<br>0.9±0.3	0.171	NA	0.055	0.157	0-1<br>0.3±0.5	0-1<br>0.5±0.5	**<0.001**	**<0.001**	**<0.001**	0.182	**<0.001**	**<0.001**	**0.035**
**Achilles tendon reflex (0-4)**	2-2<br>2.0±0.0	1-2<br>1.7±0.5	1-2<br>1.7±0.5	**<0.001**	**<0.001**	**0.001**	2-2<br>2.0±0.0	1-2<br>1.9±0.3	0.171	NA	0.055	0.157	1-2<br>1.3±0.5	1-2<br>1.5±0.5	**<0.001**	**<0.001**	**<0.001**	0.345	**<0.001**	**<0.001**	**0.013**
**Patellar reflex (0- 4)**	2-2<br>2.0±0.0	1-2<br>1.6±0.5	1-2<br>1.8±0.4	**<0.001**	**<0.001**	**0.005**	1-2<br>1.9±0.3	1-2<br>1.9±0.3	0.091	0.166	0.055	0.582	1-2<br>1.3±0.5	1-2<br>1.6±0.5	**<0.001**	**<0.001**	**<0.001**	0.085	**<0.001**	**<0.001**	0.087
**MMT (0-5) RF R**	5-5<br>5.0±0.0	3-5<br>4.3±0.8	4-5<br>4.6±0.5	**<0.001**	**<0.001**	**<0.001**	4-5<br>4.9±0.3	4-5<br>4.9±0.3	0.091	0.166	0.055	0.582	3-4<br>3.5±0.5	4-5<br>4.3±0.5	**<0.001**	**<0.001**	**<0.001**	**0.004**	**<0.001**	**<0.001**	**0.001**
**MMT (0-5) RF L**	5-5<br>5.0±0.0	4-5<br>4.2±0.4	4-5<br>4.8±0.4	**<0.001**	**<0.001**	**0.010**	4-5<br>4.3±0.5	4-5<br>4.8±0.4	**<0.001**	**<0.001**	**0.017**	**<0.001**	4-5<br>4.2±0.4	4-5<br>4.8±0.4	**<0.001**	**<0.001**	**0.006**	**0.004**	0.622	0.818	0.713
**MMT (0-5) TA R**	5-5<br>5.0±0.0	2-5<br>3.7±0.7	4-5<br>4.6±0.5	**<0.001**	**<0.001**	**<0.001**	4-5<br>4.3±0.5	4-5<br>4.9±0.3	**<0.001**	**<0.001**	0.055	**<0.001**	2-4<br>3.1±0.5	4-5<br>4.2±0.4	**<0.001**	**<0.001**	**<0.001**	**<0.001**	**<0.001**	**<0.001**	**<0.001**
**MMT (0-5) TA L**	5-5<br>5.0±0.0	4-5<br>4.4±0.5	3-5<br>4.4±0.6	**<0.001**	**<0.001**	**<0.001**	4-5<br>4.6±0.5	3-5<br>4.6±0.6	**<0.001**	**<0.001**	**0.002**	**0.0471**	4-5<br>4.1±0.4	4-5<br>4.1±0.3	**<0.001**	**<0.001**	**<0.001**	0.586	**<0.001**	**0.017**	**0.003**

Abbreviations: NTP – patients not treated with physiotherapy; TP – patients treated with physiotherapy; R – right; L – left; VAS – visual analog scale; SLR – straight leg rISe test; FvF – L3-S1 dermatome sensory perception von Frey filament test (Semmens–Weinstein test), 0 – analgesia, 0.5 – decreased, 1 – preserved, 1 – normal, 2 – hyperalgesia; Achilles and patellar tendon reflexes: 0 – absent, 1 – diminished, 2 – normal, 3 – slightly hyperactive, 4 – hyperactive with clonus; MMT – manual muscle testing (Lovett's scale), Grade 5 – normal, Grade 4 – good, Grade 3 – fair, Grade 2 – poor, Grade 1 – residual, Grade 0 – no muscle contraction; NA – not applicable.

Table S2. Comparison of electromyography recordings results from healthy volunteers and two groups of patients, treated or not treated with physiotherapy. Range, mean, and standard deviation values are presented; p≤0.05 determines significant statistical differences (marked in bold).

				sEMG results																			
				All patients					Lenke 1 group						Lenke 3 group								
**Examined muscle**	**Test**	**Side**	**Healthy volunteers (Control)**	NTP	TP	All patients vs. Control *p*	NTP patients vs. Control *p*	TP patients vs. Control *p*	NTP	TP	Lenke 1 vs. Control *p*	NTP Lenke 1 vs. Control *p*	TP Lenke 1 vs. Control *p*	NTP Lenke 1 vs. TP Lenke 1 *p*	NTP	TP	Lenke 3 vs. Control *p*	NTP Lenke 3 vs. Control *p*	TP Lenke 3 vs. Control *p*	NTP Lenke 3 vs. TP Lenke 3 *p*	Lenke 1 vs. Lenke 3 *p*	NTP Lenke 1 vs. Lenke 3 *p*	TP Lenke 1 vs. Lenke 3 *p*
**ES T5-T9**	**mcsEMG Amplitude (µV)**	R	400-800<br>592.2±22.4	100-500<br>335.0±106.8	200-600<br>420.0±90.6	**<0.001**	**<0.001**	**<0.001**	100-500<br>387.5±104.1	200-600<br>455.9±89.9	**<0.001**	**<0.001**	**<0.001**	**0.040**	200-600<br>420.0±90.6	300-550<br>373.1±69.6	**<0.001**	**<0.001**	**<0.001**	**0.001**	**<0.001**	**0.002**	**0.004**
		L	400-700<br>588.4±20.3	50-400<br>216.7±106.1	200-550<br>358.3±84.2	**<0.001**	**<0.001**	**<0.001**	100-400<br>281.3±85.4	200-550<br>385.3±84.3	**<0.001**	**<0.001**	**<0.001**	**0.001**	200-550<br>358.3±84.2	200-500<br>323.1±72.5	**<0.001**	**<0.001**	**<0.001**	**<0.001**	**<0.001**	**<0.001**	**0.043**
	**FI (4-0)**	R	2-3<br>2.9±0.4	2-3<br>2.5±0.5	2-3<br>3.0±0.2	**0.049**	**0.016**	**0.048**	2-3<br>2.9±0.3	3-3<br>3.0±0.0	0.102	0.539	NA	NA	2-3<br>3.0±0.2	2-3<br>2.9±0.3	**0.013**	**<0.001**	0.331	**<0.001**	**<0.001**	**<0.001**	NA
		L	2-3<br>2.9±0.5	1-3<br>2.2±0.5	2-3<br>2.5±0.5	**<0.001**	**<0.001**	**0.014**	2-3<br>2.4±0.5	2-3<br>2.7±0.5	**0.015**	**0.002**	0.318	0.063	2-3<br>2.5±0.5	2-3<br>2.3±0.5	**<0.001**	**<0.001**	**<0.001**	**0.022**	**0.001**	**0.009**	**0.035**
**ES T10-L2**	**mcsEMG Amplitude (µV)**	R	500-900<br>644.5 ± 27.2	50-400<br>195.0±93.2	200-500<br>373.3±76.3	**<0.001**	**<0.001**	**<0.001**	100-400<br>246.9±76.3	300-500<br>420.6±50.2	**<0.001**	**<0.001**	**<0.001**	**<0.001**	200-500<br>373.3±76.3	200-450<br>311.5±58.3	**<0.001**	**<0.001**	**<0.001**	**<0.001**	**<0.001**	**<0.001**	**<0.001**
		L	400-800<br>622.3±25.4	100-400<br>321.7±72.7	300-500<br>421.7±55.2	**<0.001**	**<0.001**	**<0.001**	100-400<br>337.5±76.4	350-500<br>444.1±49.6	**<0.001**	**<0.001**	**<0.001**	**<0.001**	300-500<br>421.7±55.2	300-500<br>392.3±49.4	**<0.001**	**<0.001**	**<0.001**	**<0.001**	**0.016**	0.087	**0.011**
	**FI (4-0)**	R	2-3<br>2.9±0.6	1-3<br>2.2±0.7	2-3<br>2.6±0.5	**<0.001**	**<0.001**	0.055	2-3<br>2.6±0.5	2-3<br>2.9±0.2	0.914	0.208	0.203	**0.031**	2-3<br>2.6±0.5	2-3<br>2.1±0.3	**<0.001**	**<0.001**	**<0.001**	**0.028**	**<0.001**	**<0.001**	**<0.001**
		L	2-3<br>2.9±0.4	2-3<br>2.7±0.5	2-3<br>3.0±0.2	0.843	0.357	0.091	2-3<br>2.9±0.3	3-3<br>3.0±0.0	0.070	0.333	NA	NA	2-3<br>3.0±0.2	2-3<br>2.9±0.3	0.253	**0.023**	0.454	0.020	**0.005**	**0.009**	NA
**RF**	**mcsEMG Amplitude (µV)**	R	700-1600<br>1410.9±105.4	550-1100<br>765.0±129.4	500-1100<br>845.0±152.2	**<0.001**	**<0.001**	**<0.001**	600-1100<br>806.3±141.3	650-1100<br>905.9±148.8	**<0.001**	**<0.001**	**<0.001**	0.058	500-1100<br>845.0±152.2	500-1000<br>765.4±119.7	**<0.001**	**<0.001**	**<0.001**	0.271	**0.004**	0.061	0.010
		L	650-1600<br>1405.2±101.1	450-1050<br>641.7±130.0	500-1100<br>771.7±148.3	**<0.001**	**<0.001**	**<0.001**	500-1050<br>643.8±156.9	500-1000<br>794.2±148.8	**<0.001**	**<0.001**	**<0.001**	**0.007**	500-1100<br>771.7±148.3	550-1100<br>742.3±148.4	**<0.001**	**<0.001**	**<0.001**	**0.041**	0.529	0.555	0.351
	**FI (4-0)**	R	2-3<br>2.9±0.3	2-3<br>2.9±0.4	2-3<br>3.0±0.2	**<0.001**	**<0.001**	**<0.001**	2-3<br>2.8±0.5	3-3<br>3.0±0.0	**<0.001**	0.070	NA	NA	2-3<br>3.0±0.2	2-3<br>2.9±0.3	**<0.001**	NA	**0.006**	NA	0.251	NA	NA
		L	2-3<br>2.8±0.5	2-3<br>2.5±0.51	2-3<br>2.7±0.5	**0.014**	**0.003**	0.181	2-3<br>2.3±0.5	2-3<br>2.8±0.4	**0.003**	**<0.001**	0.386	**0.004**	2-3<br>2.7±0.5	2-3<br>2.7±0.5	0.238	0.512	0.069	0.609	0.144	**0.004**	0.587
**TA**	**mcsEMG Amplitude (µV)**	R	600-2350<br>1768.7±125.4	600-900<br>743.3±84.8	700-1100<br>845.1±95.7	**<0.001**	**<0.001**	**<0.001**	600-900<br>762.5±86.6	700-1000<br>829.7±76.8	**<0.001**	**<0.001**	**<0.001**	**0.025**	700-1100<br>845.1±95.7	700-1100<br>865.4±116.2	**<0.001**	**<0.001**	**<0.001**	**<0.001**	0.495	0.191	0.319
		L	650-2500<br>1785.1±90.1	600-1000<br>851.7±88.6	700-1200<br>938.5±119.0	**<0.001**	**<0.001**	**<0.001**	600-1000<br>881.3±104.7	700-1100<br>920.9±109.7	<0.001	<0.001	<0.001	0.312	700-1200<br>938.5±119.0	800-1200<br>961.5±131.0	**<0.001**	**<0.001**	**<0.001**	**<0.001**	0.123	**0.015**	0.759
	**FI (4-0)**	R	3-3<br>3.0	2-3<br>2.5±0.5	2-3<br>2.7±0.5	NA	NA	NA	2-3<br>2.6±0.5	2-3<br>2.7±0.5	NA	NA	NA	0.643	2-3<br>2.7±0.5	2-3<br>2.7±0.5	NA	NA	NA	0.041	0.154	0.071	0.958
		L	3-3<br>3.0	2-3<br>2.9±0.3	3-3<br>3.0±0.0	NA	NA	NA	3-3<br>3.0±0.0	3-3<br>3.0±0.0	NA	NA	NA	NA	3-3<br>3.0±0.0	3-3<br>3.0±0.0	NA	NA	NA	NA	NA	NA	NA
**EDB**	**mcsEMG Amplitude (µV)**	R	900-4250<br>2075.6±155.2	300-700<br>521.7±110.4	500-708<br>611.9±71.9	**<0.001**	**<0.001**	**<0.001**	500-700<br>600.0±70.7	500-708<br>615.2±70.8	**<0.001**	**<0.001**	**<0.001**	**0.444**	500-708<br>611.9±71.9	500-700<br>607.7±76.0	**<0.001**	**<0.001**	**<0.001**	**<0.001**	**0.001**	**<0.001**	0.737
		L	850-4100<br>2025.1±137.1	400-900<br>671.6±134.3	600-900<br>753.6±72.8	**<0.001**	**<0.001**	**<0.001**	600-900<br>765.63±92.59	600-900<br>762.35±71.3	**<0.001**	**<0.001**	**<0.001**	**0.910**	600-900<br>753.67±72.8	600-900<br>742.3±76.1	**<0.001**	**<0.001**	**<0.001**	**<0.001**	**<0.001**	**<0.001**	0.465
	**FI (4-0)**	R	3-3<br>3.0	2-3<br>2.4±0.5	2-3<br>2.60±0.5	NA	NA	NA	2-3<br>2.56±0.51	2-3<br>2.71±0.4	NA	NA	NA	0.412	2-3<br>2.60±0.5	2-3<br>2.5±0.5	NA	NA	NA	0.369	0.043	0.140	NA
		L	3-3<br>3.0	2-3<br>2.8±0.4	2-3<br>2.80±0.4	NA	NA	NA	3-3<br>3.0±0.0	2-3<br>2.9±0.2	NA	NA	NA	NA	2-3<br>2.8±0.4	2-3<br>2.6±0.5	NA	NA	NA	0.908	<0.001	NA	NA

Abbreviations: NTP – patients not treated with physiotherapy; TP – patients treated with physiotherapy; R – right; L– left; ES – erector spinae; RF – rectus femoris; TA – tibialis anterior; EDB – extensor digiti brevis; FI – frequency index (4–0)—frequency of motor unit action potential recruitment during maximal contraction sEMG recording: 4 = >95 Hz – moderate abnormality (myogenic) 3 = 95–70 Hz—normal; 2 = 65–40 Hz—moderate abnormality (neurogenic); 1 = 35–10 Hz—severe abnormality (neurogenic); 0 = <5Hz – no contraction; NA – not applicable.

Table S3. Comparison of motor-evoked potential recording results from healthy volunteers and two groups of patients, treated or not treated with physiotherapy. Range, mean, and standard deviation values are presented; p≤0.05 determines significant statistical differences (marked in bold).

				Patient groups																			
				All patients					Lenke 1						Lenke 3						Lenke 1 vs 3		
**Recorded muscle**	**Test**	**Side**	**Healthy volunteers (Control)**	NTP	TP	All patients vs. Control *p*	NTP patients vs. Control *p*	TP patients vs. Control *p*	NTP	TP	Lenke 1 vs. Control *p*	NTP Lenke 1 vs. Control *p*	TP Lenke 1 vs. Control *p*	NTP Lenke 1 vs. TP Lenke 1 *p*	NTP	TP	Lenke 3 vs. Control *p*	NTP Lenke 3 vs. Control *p*	TP Lenke 3 vs. Control *p*	NTP Lenke 3 vs. TP Lenke 3 *p*	Lenke 1 vs. Lenke 3 *p*	NTP Lenke 1 vs. Lenke 3 *p*	TP Lenke 1 vs. Lenke 3 *p*
**RF**	**MEP Amplitude (μV)**	R	1050-2250<br>1525.0±353.3	350-500<br>426.7±36.5	400-600<br>471.7±44.9	**<0.001**	**<0.001**	**<0.001**	400-500<br>450.0±25.8	450-600<br>488.2±41.6	**<0.001**	**<0.001**	**<0.001**	**0.004**	350-450<br>400.0±27.7	400-500<br>450.0±40.8	**<0.001**	**<0.001**	**<0.001**	**0.002**	**<0.001**	**<0.001**	**0.030**
		L	1000-2100<br>1498.3±350.7	300-400<br>371.7±31.3	350-450<br>408.0±29.8	**<0.001**	**<0.001**	**<0.001**	350-400<br>390.6±20.2	400-450<br>417.7±24.6	**<0.001**	**<0.001**	**<0.001**	**0.003**	300-400<br>350.0±27.7	350-450<br>395.4±32.1	**<0.001**	**<0.001**	**<0.001**	**0.001**	**<0.001**	**<0.001**	**0.037**
	**MEP Latency (ms)**	R	19-24<br>21.9±1.1	22.2-25.2<br>23.5±0.9	22.1-24.9<br>23.5±1.0	**<0.001**	**<0.001**	**<0.001**	22.2-23.6<br>22.8±0.4	22.1-24.6<br>22.9 ± 0.7	**<0.001**	**<0.001**	**0.003**	0.856	23.6-25.2<br>24.3±0.4	23.7-24.9<br>24.4±0.5	**<0.001**	**<0.001**	**<0.001**	0.464	**<0.001**	**<0.001**	**<0.001**
		L	20-24<br>22.1±1.1	22.6-26.2<br>24.0±0.9	22.3-25.1<br>23.8±1.0	**<0.001**	**<0.001**	**<0.001**	22.6-24.5<br>23.3±0.5	22.3-24.9<br>23.1 ± 0.7	**<0.001**	**<0.001**	**0.017**	0.169	23.7-26.2<br>24.8±0.6	23.8-25.1<br>24.6±0.5	**<0.001**	**<0.001**	**<0.001**	0.770	**<0.001**	**<0.001**	**<0.001**
**TA**	**MEP Amplitude (μV)**	R	1250-2950<br>1688.3±422.2	300-600<br>423.3±74.0	300-600<br>428.3±84.8	**<0.001**	**<0.001**	**<0.001**	400-600<br>465.6±53.9	350-600<br>467.7±80.9	**<0.001**	**<0.001**	**<0.001**	0.794	300-500<br>375.0±64.3	300-450<br>376.9±59.9	**<0.001**	**<0.001**	**<0.001**	0.856	**<0.001**	**<0.001**	**0.006**
		L	1200-2900<br>1638.3±426.6	200-750<br>475.0±161.8	250-700<br>410.0±102.9	**<0.001**	**<0.001**	**<0.001**	450-750<br>609.4±77.9	300-700<br>450.0±110.4	**<0.001**	**<0.001**	**<0.001**	**<0.001**	200-450<br>321.4±61.1	250-500<br>357.7±64.1	**<0.001**	**<0.001**	**<0.001**	0.145	**<0.001**	**<0.001**	**0.012**
	**MEP Latency (ms)**	R	26-31<br>29.2±1.2	32.6-34.2<br>33.4±0.5	32.1-34.6<br>33.2±0.9	**<0.001**	**<0.001**	**<0.001**	32.8-34.2<br>33.5±0.4	32.1-32.8<br>32.5 ± 0.2	**<0.001**	**<0.001**	**<0.001**	**<0.001**	32.6-34.1<br>33.4±0.5	33.2-34.6<br>34.1±0.4	**<0.001**	**<0.001**	**<0.001**	<0.001	**<0.001**	**0.386**	**<0.001**
		L	27-31<br>29.6±1.1	32.1-33.5<br>32.9±0.4	30.0-34.3<br>32.8±1.0	**<0.001**	**<0.001**	**<0.001**	32.2-33.5<br>32.9±0.4	30.0-32.5<br>32.0 ± 0.6	**<0.001**	**<0.001**	**<0.001**	**<0.001**	32.1-33.5<br>32.8±0.4	32.5-34.3<br>33.8±0.5	**<0.001**	**<0.001**	**<0.001**	<0.001	**<0.001**	**0.269**	**<0.001**
**EDB**	**MEP Amplitude (μV)**	R	900-1950<br>1530.0±255.5	300-600<br>436.7±98.2	200-700<br>423.3±102.3	**<0.001**	**<0.001**	**<0.001**	300-600<br>496.9±80.6	300-700<br>464.7±94.8	**<0.001**	**<0.001**	**<0.001**	0.303	300-500<br>367.9±66.8	200-500<br>369.2±87.9	**<0.001**	**<0.001**	**<0.001**	0.766	**<0.001**	**<0.001**	**0.009**
		L	850-1800<br>1476.7±258.6	250-500<br>366.7±75.8	100-600<br>373.3±104.8	**<0.001**	**<0.001**	**<0.001**	300-500<br>400.0±70.7	300-600<br>414.7±89.7	**<0.001**	**<0.001**	**<0.001**	0.743	250-450<br>328.6±64.2	100-450<br>319.2±101.1	**<0.001**	**<0.001**	**<0.001**	0.926	**<0.001**	**0.009**	**0.027**
	**MEP Latency (ms)**	R	37-40<br>38.2±1.0	34.7-36.8<br>35.9±0.6	34.4-36.7<br>35.3±0.7	**<0.001**	**<0.001**	**<0.001**	34.7-36.1<br>35.4±0.4	34.4-35.4<br>34.8 ± 0.3	**<0.001**	**<0.001**	**<0.001**	**<0.001**	35.3-36.8<br>36.3±0.4	34.7-36.7<br>36.0±0.6	**<0.001**	**<0.001**	**<0.001**	0.156	**<0.001**	**<0.001**	**<0.001**
		L	37-41<br>39.1±1.3	34.0-36.2<br>35.2±0.6	34.2-36.5<br>35.1±0.7	**<0.001**	**<0.001**	**<0.001**	34.0-35.7<br>34.8±0.5	34.2-35.0<br>34.6 ± 0.3	**<0.001**	**<0.001**	**<0.001**	0.100	34.9-36.2<br>35.6±0.3	34.6-36.5<br>35.7±0.6	**<0.001**	**<0.001**	**<0.001**	0.619	**<0.001**	**<0.001**	**0.003**
**PER**	**MEP Amplitude (μV)**	R	450-2100<br>688.3±315.3	150-350<br>218.3±51.7	100-400<br>231.7±60.9	**<0.001**	**<0.001**	**<0.001**	200-350<br>246.9±46.4	150-300<br>220.6±43.5	**<0.001**	**<0.001**	**<0.001**	0.130	150-250<br>185.7±36.3	100-400<br>246.2±77.6	**<0.001**	**<0.001**	**<0.001**	**0.017**	0.127	**<0.001**	0.249
		L	400-2100<br>655.0±308.9	100-300<br>171.7±52.0	100-300<br>186.7±60.1	**<0.001**	**<0.001**	**<0.001**	100-300<br>190.6±49.1	100-300<br>182.4±46.6	**<0.001**	**<0.001**	**<0.001**	0.577	100-250<br>150.0±48.0	100-300<br>192.3±76.0	**<0.001**	**<0.001**	**<0.001**	0.134	0.112	0.019	0.879
	**MEP Latency (ms)**	R	23-29<br>25.9±1.8	27.5-29.8<br>28.6±0.7	27.2-29.9<br>28.6±0.8	**<0.001**	**<0.001**	**<0.001**	27.5-28.7<br>28.1±0.4	27.2-28.8<br>28.0 ± 0.5	**<0.001**	**<0.001**	**<0.001**	0.764	28.6-29.8<br>29.2±0.3	28.6-29.9<br>29.2±0.4	**<0.001**	**<0.001**	**<0.001**	0.986	**<0.001**	**<0.001**	**<0.001**
		L	23-30<br>26.8±2.0	27.0-28.9<br>27.9±0.6	20.0-29.7<br>28.1±1.7	**0.001**	**0.014**	**0.002**	27.0-28.1<br>27.4±0.3	27.0-28.5<br>27.8 ± 0.5	0.048	0.102	**<0.001**	**0.004**	27.9-28.9<br>28.4±0.2	20.0-29.7<br>28.4±2.6	**<0.001**	**<0.001**	**0.002**	**0.004**	**<0.001**	**<0.001**	**<0.001**

Abbreviations: NTP – patients not treated with physiotherapy; TP – patients treated with physiotherapy; R – right; L – left; RF – rectus femoris; TA – tibialis anterior; EDB – extensor digiti brevis; PER – peroneal nerve.

Table S4. Comparison of the bilateral electroneurography (ENG) recordings results following the stimulation of peroneal nerves in healthy volunteers and two groups of patients, treated or not treated with physiotherapy. Range, mean, and standard deviation values are presented; p≤0.05 determines significant statistical differences (marked in bold).

			**All patients**					**Lenke 1**						**Lenke 3**						
Parameter	Side	Healthy volunteers (Control)	NTP	TP	All patients vs. Control *p*	NTP vs. Control *p*	TP vs. Control *p*	NTP	TP	Lenke 1 vs. Control *p*	NTP Lenke 1 vs. Control *p*	TP Lenke 1 vs. Control *p*	NTP Lenke 1 vs. TP Lenke 1 *p*	NTP	TP	Lenke 3 vs. Control *p*	NTP Lenke 3 vs. Control *p*	TP Lenke 3 vs. Control *p*	NTP Lenke 3 vs. TP Lenke 3 *p*	Lenke 1 vs. Lenke 3 *p*
**M-wave amplitude (μV)**	R	3100-10500<br>6383.3±203.6	1200-8400<br>3063.3±178.7	2700-9000<br>4246.7±149.9	**<0.001**	**<0.001**	**<0.001**	1900-8400<br>4106.3±186.4	3500-9000<br>5280.0±147.7	**<0.001**	**0.001**	**0.024**	0.117	1200-2800<br>1871.4±474.6	2800-4200<br>3209.1±425.3	**<0.001**	**<0.001**	**<0.001**	**<0.001**	**<0.001**
	L	3200-10000<br>6383.3±198.8	1400-9800<br>4816.7±276.7	3000-10000<br>6066.7±190.4	**<0.001**	**<0.001**	**<0.001**	2200-9800<br>6387.5±272.1	6200-10000<br>7326.7±125.5	0.265	0.995	0.101	0.493	1400-6500<br>3021.4±139.4	3000-7300<br>4754.5±452.1	**<0.001**	**<0.001**	**0.004**	**0.013**	**<0.001**
**M-wave latency (ms)**	R	3-5<br>4.2±0.5	4-6<br>4.5±0.7	4-5<br>4.5±0.4	**<0.001**	**<0.001**	**<0.001**	4-6<br>4.6±0.7	4-5<br>4.3±0.4	0.212	0.087	0.703	0.187	4-6<br>4.5±0.7	4-5<br>4.7±0.4	**0.045**	0.276	**0.006**	0.293	0.241
	L	3-6<br>4.4±0.5	4-6<br>4.9±0.6	4-6<br>4.8±0.5	**<0.001**	**<0.001**	**<0.001**	4-6<br>4.9±0.6	4-5<br>4.7±0.5	**0.004**	**0.006**	0.058	0.312	4-6<br>4.9±0.7	4-6<br>4.9±0.5	**0.003**	**0.017**	**0.014**	0.914	0.636
**F-wave frequency**	R	15-20<br>17.6±1.6	11-18<br>14.6±2.0	13-20<br>16.9±1.9	**<0.001**	**<0.001**	**<0.001**	12-18<br>15.3±1.7	15-20<br>17.5±1.6	**0.008**	**<0.001**	0.558	**<0.001**	11-18<br>13.9±2.1	13-20<br>16.1±2.2	**<0.001**	**<0.001**	0.094	**0.009**	**0.035**
	L	14-20<br>17.0±1.5	14-19<br>16.4±1.5	14-20<br>18.0±1.6	**<0.001**	**<0.001**	**<0.001**	15-19<br>16.9±1.4	16-20<br>18.1±1.2	0.123	0.843	**0.009**	**0.015**	14-19<br>15.8±1.4	14-20<br>17.5±2.0	0.645	**0.020**	0.130	**0.012**	0.140

Abbreviations: NTP – patients not treated with physiotherapy; TP – patients treated with physiotherapy; R – right; PER – peroneal nerve.
